# Preface

**DOI:** 10.3897/zookeys.1044.69727

**Published:** 2021-06-16

**Authors:** John Spence, Achille Casale, Thorsten Assmann, James K. Liebherr, Lyubomir Penev

**Affiliations:** 1 University of Alberta, Edmonton, Canada University of Alberta Edmonton Canada; 2 National Parks Service, Page, USA National Parks Service Page United States of America; 3 Corso Raffaello 12, 10126 Torino, Italy University of Sassari, Italy (Zoology). Private: Corso Raffaello 12, 10126 Torino Italy; 4 Institute of Ecology, Leuphana University Lüneburg, Universitätsstraße 1, 21335, Lüneburg, Germany Leuphana University Luneburg Luneburg Germany; 5 Department of Entomology, John H. and Anna B. Comstock Hall, 129 Garden Ave., Cornell University, Ithaca, NY 14853-2601, USA Cornell University Ithaca United States of America; 6 Institute of Biodiversity & Ecosystem Research – Bulgarian Academy of Sciences, Sofia, Bulgaria Institute of Biodiversity & Ecosystem Research – Bulgarian Academy of Sciences Sofia Bulgaria; 7 Pensoft Publishers, Sofia, Bulgaria Pensoft Publishers Sofia Bulgaria

## Abstract

This special issue of ZooKeys celebrates the memory of Dr. Terry Lee Erwin (1940-2020), who was the founding Editor of this journal. Terry was also a preeminent systematist specializing in the beetle family Carabidae, as well as one of the world’s principal authorities on the biodiversity crisis, especially in relation to the canopy fauna of tropical forests. His contributions as a practicing scientist, an educator and a public advocate for insect natural history were enormous. His unexpected passing in May 2020 was a significant loss to students of the Carabidae and to the worldwide community interested in understanding the biological diversity of Neotropical forests. In this volume we showcase his influence on others, both through a set of scientific papers that reflect the breadth of that influence, and a series of personal memories offered by people who worked and interacted with Terry. That influence includes both the direct impacts of his own scientific work and the unusual level of encouragement and generous support that he provided to others who pursued work on carabid beetles. The volume begins with a short biography of Dr. Erwin, emphasizing his connections to the New World tropics and the genesis of his work on the beetles that live there, followed by a general summary of his scientific accomplishments and the legacy that he leaves behind to support and encourage work in the areas that he touched. The work closes with a view of his most central work as a taxonomist by listing the genera and species that he described, and the taxa named in his honour by others in recognition of his influence on the field. This volume was made possible through the generous support of Pensoft Publishers. The Editors sincerely thank all contributors and the highly competent staff of ZooKeys for their efforts in producing this memorial to our friend, colleague and exemplary systematist.

This special issue of ZooKeys celebrates the memory of Dr. Terry Lee Erwin (1940–2020), who was the founding Editor of this journal. Terry was also a preeminent systematist specializing in the beetle family Carabidae, as well as one of the world’s principal authorities on the biodiversity crisis, especially in relation to the canopy fauna of tropical forests. His contributions as a practicing scientist, an educator and a public advocate for insect natural history were enormous. His unexpected passing in May 2020 was a significant loss to students of the Carabidae and to the worldwide community interested in understanding the biological diversity of Neotropical forests. In this volume we showcase his influence on others, both through a set of scientific papers that reflect the breadth of that influence, and a series of personal memories offered by people who worked and interacted with Terry. That influence includes both the direct impacts of his own scientific work and the unusual level of encouragement and generous support that he provided to others who pursued work on carabid beetles. The volume begins with a short biography of Dr. Erwin, emphasizing his connections to the New World tropics and the genesis of his work on the beetles that live there, followed by a general summary of his scientific accomplishments and the legacy that he leaves behind to support and encourage work in the areas that he touched. The work closes with a view of his most central work as a taxonomist by listing the genera and species that he described, and the taxa named in his honour by others in recognition of his influence on the field. This volume was made possible through the generous support of Pensoft Publishers. The Editors sincerely thank all contributors and the highly competent staff of ZooKeys for their efforts in producing this memorial to our friend, colleague and exemplary systematist.

**John R. Spence**

Edmonton, Alberta, Canada

**Achille Casale**

Torino, Italy

**Thorsten Assmann**

Lüneburg, Germany

**James K. Liebherr**

Ithaca, NY, USA

**Lyubomir Penev**

Sofia, Bulgaria

